# A Splice Isoform of DNedd4, DNedd4-Long, Negatively Regulates Neuromuscular Synaptogenesis and Viability in *Drosophila*

**DOI:** 10.1371/journal.pone.0027007

**Published:** 2011-11-14

**Authors:** Yunan Zhong, Alina Shtineman-Kotler, Leo Nguyen, Konstantin G. Iliadi, Gabrielle L. Boulianne, Daniela Rotin

**Affiliations:** 1 Program in Cell Biology, The Hospital for Sick Children, Toronto, Canada; 2 Program in Developmental and Stem Cell Biology, The Hospital for Sick Children, Toronto, Canada; 3 Department of Biochemistry, University of Toronto, Toronto, Canada; 4 Department of Molecular Genetics, University of Toronto, Toronto, Canada; Oregon Health and Science University, United States of America

## Abstract

**Background:**

Neuromuscular (NM) synaptogenesis is a tightly regulated process. We previously showed that in flies, *Drosophila* Nedd4 (dNedd4/dNedd4S) is required for proper NM synaptogenesis by promoting endocytosis of commissureless from the muscle surface, a pre-requisite step for muscle innervation. DNedd4 is an E3 ubiquitin ligase comprised of a C2-WW(x3)-Hect domain architecture, which includes several splice isoforms, the most prominent ones are dNedd4-short (dNedd4S) and dNedd4-long (dNedd4Lo).

**Methodology/Principal Findings:**

We show here that while dNedd4S is essential for NM synaptogenesis, the dNedd4Lo isoform inhibits this process and causes lethality. Our results reveal that unlike dNedd4S, dNedd4Lo cannot rescue the lethality of dNedd4 null (*DNedd4^T121FS^*) flies. Moreover, overexpression of UAS-*dNedd4Lo* specifically in wildtype muscles leads to NM synaptogenesis defects, impaired locomotion and larval lethality. These negative effects of dNedd4Lo are ameliorated by deletion of two regions (N-terminus and Middle region) unique to this isoform, and by inactivating the catalytic activity of dNedd4Lo, suggesting that these unique regions, as well as catalytic activity, are responsible for the inhibitory effects of dNedd4Lo on synaptogenesis. In accord with these findings, we demonstrate by sqRT-PCR an increase in dNedd4S expression relative to the expression of dNedd4Lo during embryonic stages when synaptogenesis takes place.

**Conclusion/Significance:**

Our studies demonstrate that splice isoforms of the same dNedd4 gene can lead to opposite effects on NM synaptogenesis.

## Introduction

Neuronal precursor cell-expressed developmentally downregulated 4 (Nedd4) is an E3 ubiquitin ligase that belongs to the Hect family [1]. Nedd4 proteins share common domain architecture: a C2 domain, 3–4 WW domains and a ubiquitin ligase Hect domain. The C2 domain is primarily responsible for sub-cellular targeting [2], [3], while the WW domains mediate substrate recognition and binding usually by associating with PY motifs (L/PPxY) [4], [5], [6].

In higher eukaryotes, there are several Nedd4 family proteins, including the closely related Nedd4-1 (Nedd4) and Nedd4-2 (Nedd4L). In mammals, Nedd4-2 is known to regulate stability of ion channels, such as ENaC, which has PY motifs that interact with the Nedd4-2 -WW domains to promote ENaC endocytosis [7], [8], [9], [10]. Mutations in ENaC PY motifs found in Liddle syndrome (a hereditary hypertension) result in increased retention of ENaC at the plasma membrane in the kidney [11], [12]. The interaction between ENaC and Nedd4-2 can be negatively regulated through the phosphorylation of Nedd4-2 by the Ser/Thr kinase, Sgk1, and its close relative Akt1 [13], [14]. In contrast to Nedd4-2, mammalian Nedd4-1 has been implicated in the regulation of cellular and animal growth [15], T cell activation [16] and heart [17] and nervous system [18] development.

Recently, Nedd4, which is expressed in muscles, was found to regulate neuromuscular (NM) synaptogenesis in flies [19] and mammals [20]. Specifically, *Drosophila* Nedd4 (dNedd4) was shown to regulate endocytosis of commissureless (Comm) from the muscle surface to allow proper initiation of NM synaptogenesis [19]. Comm contains two PY motifs (PPCY and LPSY) and 10 Lys residues (ubiquitin acceptor sites) in its intracellular domain. The WW domains of dNedd4 bind to the PY motifs of Comm, leading to Comm ubiquitylation [21]. In *Drosophila*, each body wall hemisegment contains 30 muscle fibers that are innervated by ∼40 motor neurons in a specific, precisely timed manner [22]. Internalization of Comm (expressed on the muscle cell surface) into the muscle is a prerequisite for proper initiation of NM synaptogenesis [23] and is facilitated by dNedd4 [19]. Of note, the dNedd4 gene is subject to alternative splicing, with several isoforms that can be generally divided into two groups represented by a short isoform (dNedd4S, or dNedd4) and a long isoform, dNedd4Lo. While dNedd4S (dNedd4) was previously shown to enhance NM synaptogenesis, the function(s) of dNedd4Lo was unknown.

Here we characterized dNedd4Lo and show that in contrast to dNedd4S, dNedd4Lo has a negative function in NM synaptogenesis, leading to defects in neuromuscular synapse formation and abnormal larval locomotion.

## Results

**Figure 1:**
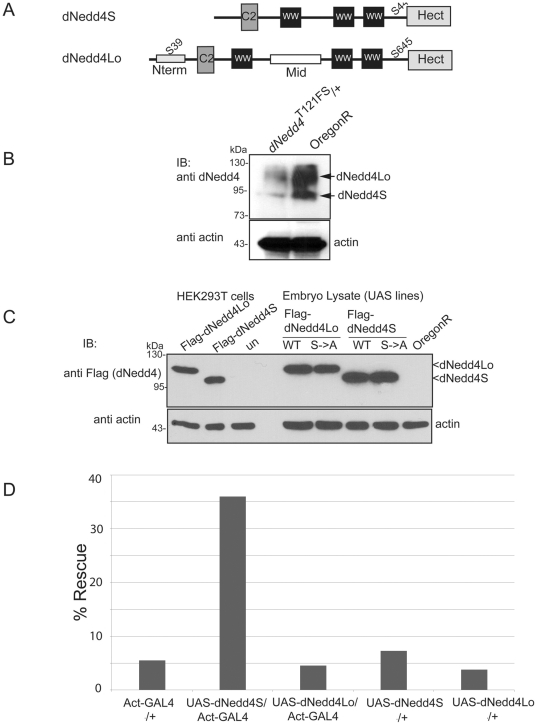
dNedd4S, but not dNedd4Lo, can rescue the lethality of dNedd4 null mutant flies. (A) Schematic representation of dNedd4S and dNedd4Lo showing their common C2, WW(x3) and Hect domains, putative Akt phosphorylation sites, and N-terminal (Nterm) and middle (Mid) regions unique to dNedd4Lo. (B) Reduction of endogenous dNedd4 protein expression in *dNedd4* ^T121FS^ heterozygote compared to *Oregon-R* wild type (WT) embryos, detected with an anti-dNedd4 antibody that recognizes both dNedd4S and dNedd4Lo. (C) Protein expression of Flag-tagged UAS-*dNedd4Lo* WT, *dNedd4S* WT and their S->A mutants driven by *daughterless(da)*-GAL4 in embryo, detected using anti-Flag antibody on western blots (upper panel). Lower panel: actin loading controls. OregonR embryo lysate was included as a negative control, and lysates from mammalian HEK293T cells transfected with dNedd4Lo and dNedd4S were included as positive controls (upper panel). un = untransfected. (D) Expression of dNedd4S, but not dNedd4Lo, driven by *Actin*-GAL4 throughout the embryo partially rescues *dNedd4* null mutant viability. The bar chart represents the percentage of viable *dNedd4* null mutant embryos (measured as hatched larvae without GFP expression) out of the expected number of mutant embryos. 800 embryos were scored per genotype.

Our previous work showed that dNedd4 is involved in regulating neuromuscular synaptogenesis, and revealed two isofoms of dNedd4 expressed in the body wall muscle [19]. In accord, our immunoblotting analysis of embryo lysates revealed two major splice isoforms of dNedd4, which we named dNedd4-short (dNedd4S, ∼92 kDa) and dNedd4-long (dNedd4Lo, ∼112 kDa) (Fig. 1A,B). DNedd4Lo possesses the same functional domains as dNedd4S: a conserved C2 domain, 3 WW domains, including the high affinity WW3 binding domain [24] and a Hect domain, which is catalytically active, much like dNedd4S (Fig. S1A). In addition, dNedd4Lo contains a unique N-terminal (Nterm) region that includes a putative Akt phosphorylation site, as well as a unique middle (Mid) region (Fig. 1A). Our previous work focused on the function of dNedd4S [19]. Here we investigated the biological role of dNedd4Lo and compared it to that of dNedd4S.

### DNedd4S, but not dNedd4Lo, can partially rescue lethality of dNedd4 null mutant flies

To determine the biological importance of the two splice isoforms of dNedd4, we tested their ability to rescue *dNedd4* null mutant flies. The *dNedd4* null (*dNedd4* ^T121FS^ homozygote) flies contain a frame shift mutation in the dNedd4 gene that truncates the protein products of all the dNedd4 splice isoforms at Thr121, which renders dNedd4 inactive. *DNedd4* ^T121FS^ flies are heterozygous viable and homozygous lethal at the embryonic stage. For rescue experiments, flies containing either a UAS-*dNedd4S* or UAS*-dNedd4Lo* transgene under the transcriptional control of the ubiquitous *Act*-GAL4 driver [25] (Fig. 1C) were crossed into *dNedd4* ^T121FS^ homozygote flies. Two independent crosses were performed for each of the UAS-*dNedd4S* and UAS*-dNedd4Lo* transgenes, as well as their respective *Act*-GAL4 driver alone and UAS transgene alone controls. A total of 800 embryos were collected for each UAS line and observed under a fluorescent microscope to follow the survival of rescued larvae, which do not express GFP (non-GFP). Rescue was determined by measuring the percentage of viable *dNedd4* null mutant embryos (hatched larvae without GFP expression) out of the expected number of mutant embryos. The expected number of genotype of interest for rescue with dNedd4S (UAS-*dNedd4S*/X; *Act*-GAL4/CyO (or Sp); *dNedd4* ^T121FS^/*dNedd4* ^T121FS^) was calculated to be 12.5%, and with dNedd4Lo (+; UAS-*dNedd4Lo*/CyO (or Sp); *dNedd4* ^T121FS^/*dNedd4* ^T121FS^) was calculated to be 25%. This excludes all other possible genotypes. Our results show that expression of dNedd4S throughout the embryo partially rescued the lethality of *dNedd4* null embryos (from 5.5% or 7.25% to 36%), whereas dNedd4Lo did not rescue the lethality (From 5.5% or 3.75% to 4.5%) and larvae died soon after egg hatching (and some did not fully crawl out of their egg shells) (Fig. 1D). These results suggest that the two splice isoforms of dNedd4, both of which are expressed endogenously in embryos (Fig. 1B), have distinct roles during embryo development.

### Overexpression of dNedd4Lo in the muscle reduces fly viability

Given the different effects of dNedd4S and dNedd4Lo on rescuing *dNedd4* null mutant flies, we next examined the effect of overexpression of dNedd4Lo and dNedd4S. Our results show that ubiquitous expression of all transgenic lines of UAS-*dNedd4Lo* crossed with the *Act*-GAL4 driver resulted in lethality before the third instar larval stage, while flies expressing UAS-*dNedd4S* survived to the adult stage. In an attempt to observe the effect of slight variations in expression levels on fly survival, different ubiquitous GAL4 drivers, including *da*-GAL4 [26], *Actin*-GAL4 and *Tubulin*-GAL4 [27] were used at 25°C, room temp (RT, ∼22°C), or 18°C. All ubiquitous drivers at all temperatures yielded developmental lethality when crossed to UAS-*dNedd4Lo*, but not UAS-*dNedd4S* (Table S1). To rule out the possibility that lethality was due to a defect in sub-cellular localization of the protein, we examined the distribution of dNedd4Lo and dNedd4S in salivary glands. The proteins did not form aggregates and properly localized in the cytosol and on the plasma membrane (Fig. S1B), as was previously observed for localization of endogenous dNedd4 [19] [28]. In an attempt to determine the cause of lethality, various tissue-specific GAL4 drivers were used to drive overexpression in the CNS using *elav* ^c155^-GAL4 [29], in motor neurons using *D42*-GAL4 [29], in muscles using *24B*-GAL4 [29] or *5*-GAL4 [19], in eye using *GMR*-GAL4 [30], in fat body using Ppl-GAL4 [31] and in the epithelial lining of the digestive system and respiratory system using *48Y*-GAL4 [29]. Each test was performed at 25°C, RT, or 18°C, to determine whether slight variations in expression levels had any effect on fly survival. We found that all UAS-*dNedd4Lo* transgenic flies died during development only when overexpressed in muscle using the *24B*-GAL4 or *5*-GAL4 drivers, while UAS-*dNedd4S* transgenic files survived to adulthood (Table S2). Once again, the lethality was not due to a defect in protein sub-cellular localization since we found that dNedd4Lo was properly localized in the muscle (see below).

### Overexpression of UAS-*dNedd4Lo* in muscle results in aberrant synaptic innervation along the SNb branch from body wall muscle 13 to 12

**Figure 2:**
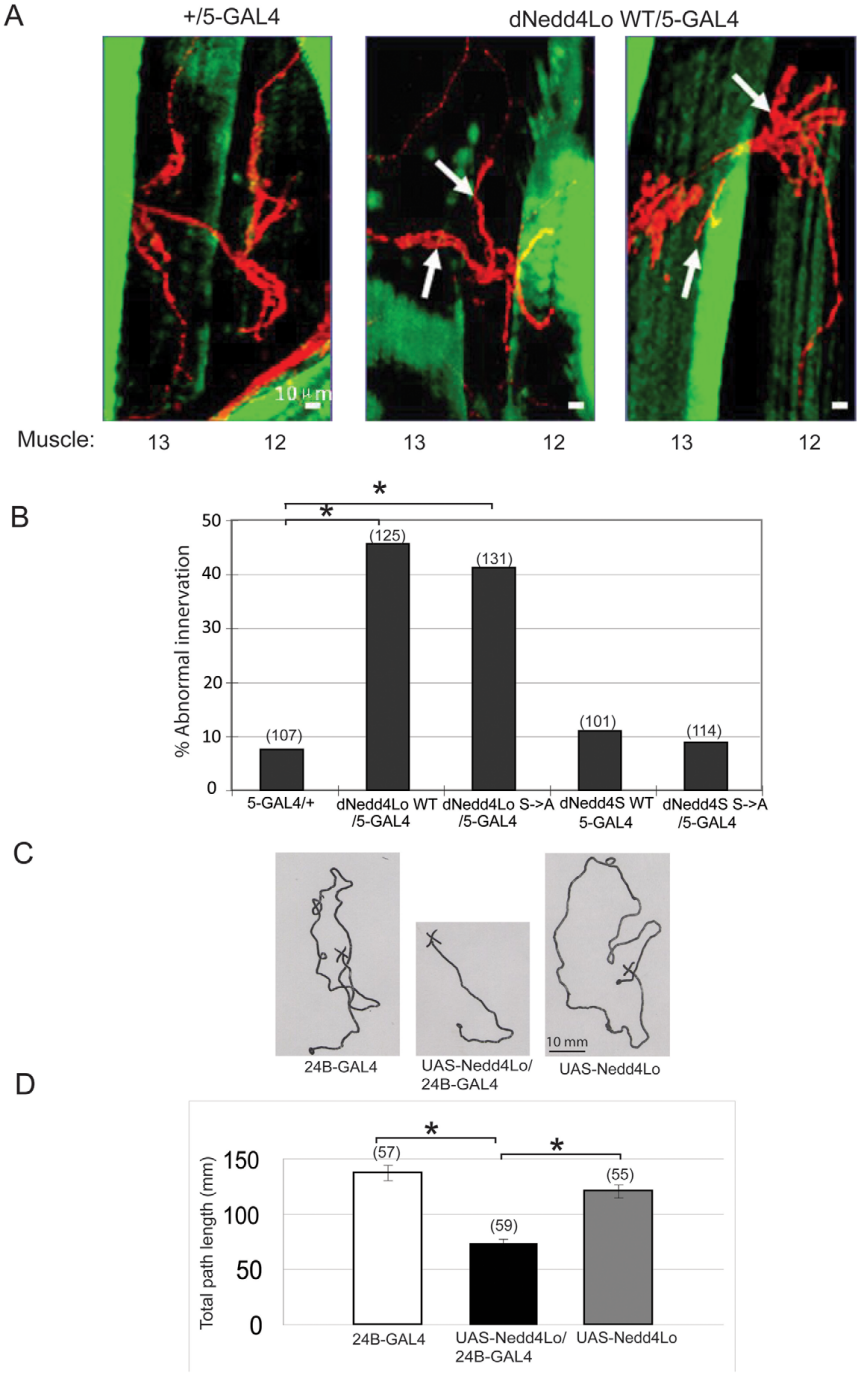
Neuromuscular innervation and locomotion defects in larvae overexpressing dNedd4Lo in the muscle. (A) Muscle-specific overexpression of UAS-*dNedd4Lo* (*dNedd4Lo*/*5*-GAL4) leads to aberrant synaptic innervation along the SNb branch from body wall muscles 13->12 of third instar larvae (HRP stain, red). Muscle driver control line (+/*5*-GAL4) was included (left panel) to show the normal motor neuron innervation pattern on muscle 13->12. Muscles were stained with Phalloidin (green). Scale bars, 10 µm. (B) Quantification of the muscle innervation defects. Numbers in brackets denote the number of muscles scored (n). * p<0.0001 (Two tailed Fisher's exact test). (C,D) Overexpression of dNedd4Lo adversely affects larval locomotor activity measured by total path length: (C) Representative path travelled by third instar larvae of muscle driver alone (*24B*-GAL4), UAS-*dNedd4Lo* alone (UAS-*dNedd4Lo*), or larvae overexpressing dNedd4Lo in the muscle (UAS-*dNedd4Lo/24B*-GAL4). Scale bar, 10 mm. (D) Quantification of locomotor activity shown in panel C of 55–60 larvae (per genotype). Numbers in brackets denote the exact number of larvae scored (n). * indicates a significant difference between the UAS-*dNedd4Lo/24B*-GAL4 line and both control lines (UAS-*dNedd4Lo* alone or *24B*-GAL4 alone) (p<0.001, post hoc analysis).

Since we previously demonstrated that dNedd4S is involved in neuromuscular (NM) synaptogenesis [19], potential defects in NM synapse formation were analyzed by performing immunofluorescence staining on dissected body wall muscles of third instar larvae from UAS-*dNedd4Lo* transgenic lines. There are two major categories of innervation defect: pathfinding error and axonal overgrowth/undergrowth error. The abnormal innervations that were identified and scored in this experiment included: backward innervation from muscle 12 onto muscle 13, which belongs to pathfinding error, and increased branching on muscle 12, which belongs to overgrowth error (Fig. 2A). The frequency of abnormalities was calculated as the number of abnormal innervations (backward innervation and/or pathfinding error) that were identified over the total number of neuromuscular junction at muscle 13 and muscle 12 that were scored. By scoring ∼100–130 NM synapses at muscles 13 and 12 from each of the UAS-*dNedd4Lo* and UAS-*dNedd4S* transgenic lines, the number of normal and abnormal innervation for each UAS-*dNedd4* transgenic was compared with that of the muscle driver control (*5*-GAL4 muscle driver fly used to drive overexpression of the UAS transgenics) (Fig. 2B). Our results show that the UAS-*dNedd4Lo* transgenics had a significant amount of abnormal innervation relative to the control (two-tailed Fisher's exact test, p<0.0001), which was not observed for the UAS-*dNedd4S* transgenics (p<0.4734).

Taken together, these results demonstrate that muscle-specific overexpression of UAS-*dNedd4Lo* in a wild type background causes a high frequency of abnormal motor neuron innervation on muscles 13 and 12, whereas that of UAS-*dNedd4S* does not cause significant abnormalities.

### Overexpression of UAS-*dNedd4Lo* in muscle results in reduction of larval locomotor activity

To determine the consequences of the abnormal muscle innervation in larvae that overexpress dNedd4Lo in the muscle, we analyzed larval locomotor activity by measuring the total path length travelled by pre-wandering third instar larvae over a 150 sec period. Our results reveal a severe reduction in locomotor activity of larvae that overexpress dNedd4Lo in the muscle (+/UAS-*dNedd4Lo*; +/*24B*-GAL4) (73.76±3.80 mm) relative to the +/UAS-*dNedd4Lo* (120.35±5.05 mm) or +/*24B*-GAL4 (137.23±5.46 mm) controls (post hoc analysis, p<0.001) (Fig. 2C,D).

### Adverse effects of dNedd4Lo are not a result of dAkt regulation

**Figure 3:**
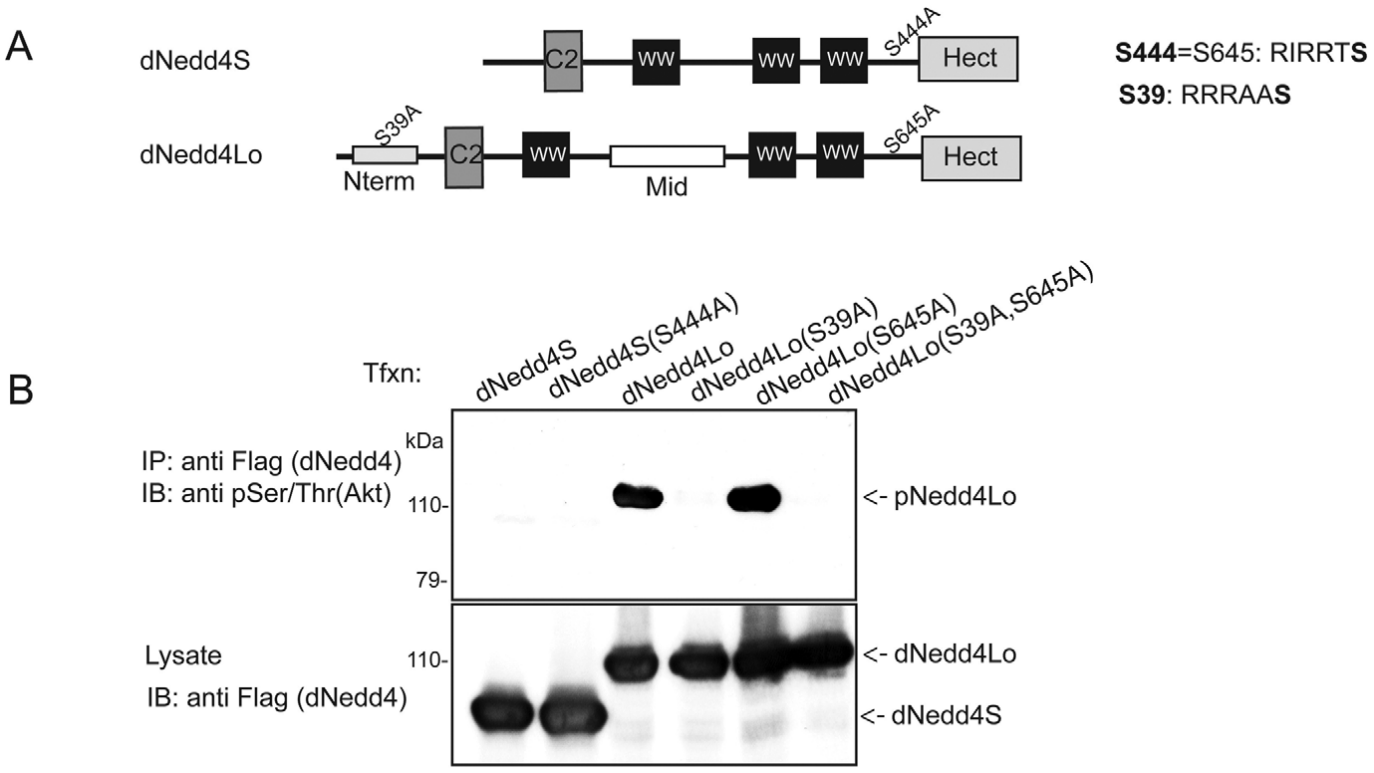
DNedd4Lo, but not dNedd4S, is phosphorylated by Akt in *Drosophila* S2 cells. (A) Schematic representation of dNedd4S and dNedd4Lo showing their consensus Akt phosphorylation sites. (B) *Drosophila* S2 cells were transfected (Tfxn) with Flag-tagged wild-type (WT) or the phosphorylation mutants of the dNedd4 isoforms: dNedd4S (dNedd4S(WT)), dNedd4S(S444A), dNedd4Lo(WT), dNedd4Lo(S39A), dNedd4Lo(S645A), or dNedd4Lo (S39A,S645A). Transfected cells were lysed and the lysates immunoprecipitated (IP) with anti Flag antibodies and immunoblotted (IB) with anti Phospho-(Ser/Thr) Akt substrate antibodies to detect phosphorylated dNedd4 (upper panel). DNedd4 expression levels in the lysates were verified with anti-Flag antibodies (lower panel).

Differences between the two isoforms of dNedd4 include an alternate start codon site resulting in a longer N-terminal region in dNedd4Lo, and an extra exon inserted between the WW1 and WW3 domains (Fig. 1A and 3A). In the unique N-terminal region of dNedd4Lo, there is a putative Akt phosphorylation site (S39) with the consensus sequence, RxRxxS/T. Another putative Akt phosphorylation site was found common to both dNedd4Lo (S645) and dNedd4S (S444) (Fig. 3A). Interestingly, a close relative of dNedd4 in mammals, Nedd4-2, also contains a consensus sequence, RxRxxS/T, that can be phosphorylated by the Ser/Thr kinase Sgk1 and its close relative, Akt1 [13]. Therefore, we tested the possibility that the putative Akt phosphorylation site(s) in dNedd4Lo were phosphorylated by *Drosophila* Akt (dAkt) in *Drosophila* S2 cells. We found that while the site containing Ser39 in the unique N-terminal region of dNedd4Lo was indeed phosphorylated, the site containing Ser653 in dNedd4Lo, which is also found in dNedd4S (Ser543), was not phosphorylated by dAkt (Fig. 3B). Ser39->Ala mutation in dNedd4Lo abolished its phosphorylation. This observation led us to ask whether dNedd4Lo's unique function is mediated by dAkt phosphorylation. To test this possibility, we generated UAS transgenic lines that express a *dNedd4Lo* S39A;S645A double mutant (UAS-*dNedd4Lo* 2S->A), in which the Ser residue of each putative Akt phosphorylation site was mutated to Ala. A UAS-*dNedd4S* S444A (UAS-*dNedd4S* S->A) mutant transgenic line was also generated for comparison (Fig. 3A). We performed the same lethality tests using ubiquitous and muscle-specific GAL4 drivers as described above for UAS-*dNedd4Lo* and UAS-*dNedd4S* (wildtype, WT). No difference was found between dNedd4Lo WT and its 2S->A mutant, nor between dNedd4S WT and its S->A mutant (Table S3). Next, we scored for muscle innervation defects from UAS-*dNedd4Lo* 2S->A and UAS-*dNedd4S* S->A transgenic flies as described above for their WT counterpart. Again, no significant difference was found between UAS-*dNedd4Lo* WT and its 2S->A mutant (p<0.5288) (Fig. 2B). Because removing the dAkt phosphorylation site of dNedd4Lo mutant did not alter the muscle innervation defects, we conclude that the negative role of dNedd4Lo in NM synaptogenesis is not due to dAkt regulation.

### Both unique sequences and the catalytic activity of Nedd4Lo are involved in its regulation of neuromuscular synaptogenesis

**Figure 4:**
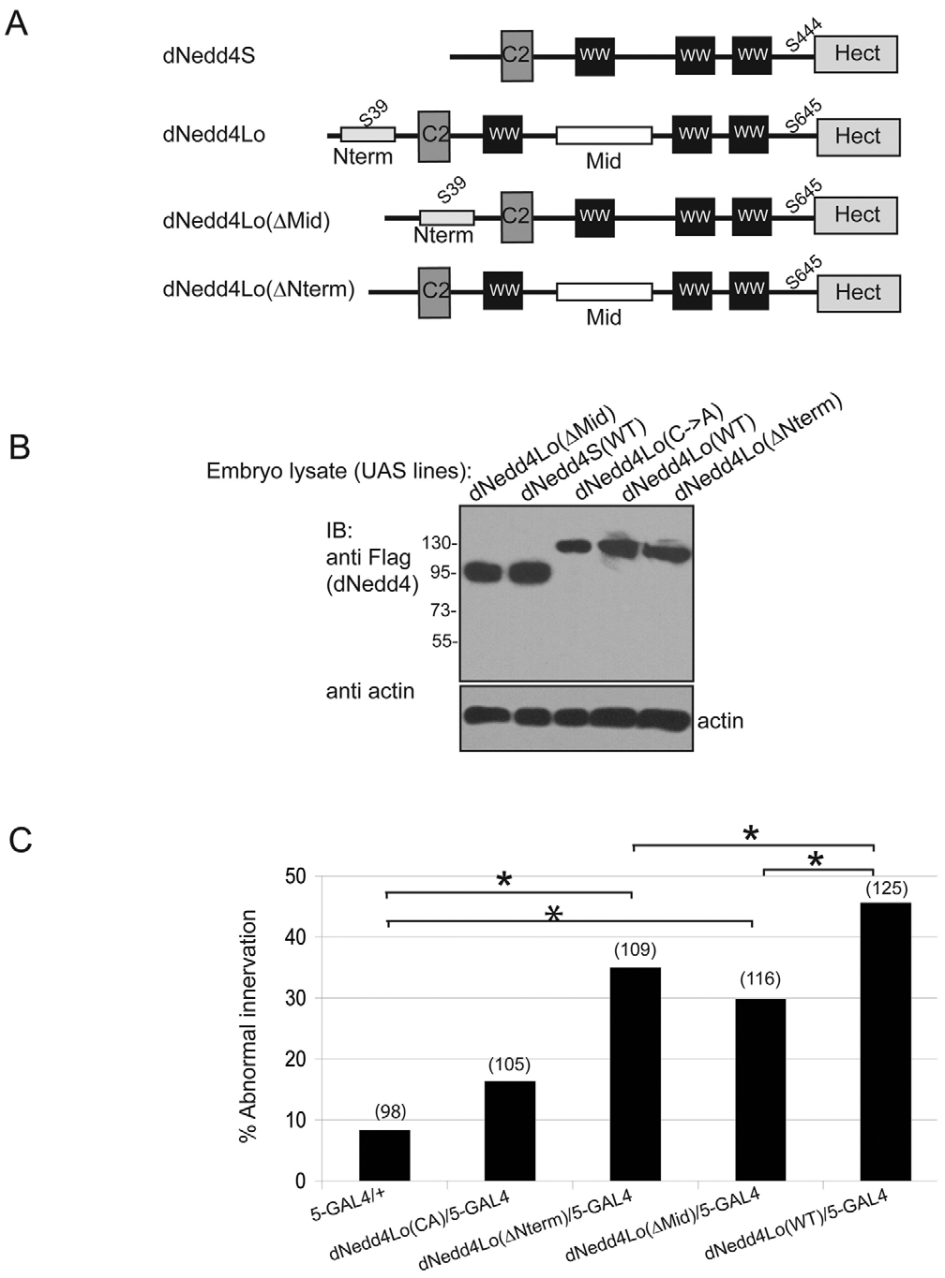
The unique N-terminus and middle regions of dNedd4Lo are required for its inhibition of NM synaptogenesis. (A) Schematic representation of dNedd4Lo mutants: dNedd4LoΔMiddle (Mid) has the middle unique sequence deleted and dNedd4LoΔN-terminus (Nterm) has the N-terminal unique sequence replaced by the one from dNedd4S. (B) Protein expression of Flag-tagged UAS-*dNedd4oL*ΔNterm, *dNedd4L*ΔMid or the catalytically–inactive *dNedd4Lo* C->A mutants driven by *da*-GAL4 in embryos, detected by immunoblotting (IB) with anti-Flag antibodies. Equal amount of lysate was loaded as shown by the actin loading control (lower panel). (C) Muscle innervation defects (scored on muscles 12 and 13) are decreased in embryos/larvae expressing dNedd4Lo lacking its Nterm, Mid region or its catalytic activity in the muscle. Numbers in brackets reflect the number of muscles scored (n). * *p*<0.03, Fisher's exact test (two tailed).

Since the dAkt phosphorylation site in the unique N-terminal region of dNedd4Lo did not explain the functional difference between dNedd4Lo and dNedd4S, we next studied the role of the N-terminal (Nterm) region as well as the unique middle (Mid) region of dNedd4Lo to determine if either region is involved in the adverse function of dNedd4Lo in NM synaptogenesis. Two dNedd4Lo deletion mutants were created: One with the middle unique sequence deleted (dNedd4LoΔMid) and the other, dNedd4LoΔNterm, had the N-terminal unique sequence replaced by the one from dNedd4S. In addition, a catalytically inactive Cys961->Ala (C->A) mutant of dNedd4Lo was created to test the effect of abolishing the ubiquitin ligase activity of dNedd4Lo on its function in NM synaptogenesis (Fig. 4 A,B). These mutants were analyzed in comparison to dNedd4Lo WT. Ubiquitous overexpression of UAS-*dNedd4L*ΔNterm crossed with the *da*-GAL4 driver, *Actin*-GAL4, or *Tubulin*-GAL4 in a wild type background at 25°C, RT or 18°C resulted in developmental lethality, while UAS-*dNedd4Lo*ΔMid and UAS-*dNedd4Lo* C->A transgenic flies survived to adulthood (Table S3). Muscle-specific overexpression of UAS-*dNedd4Lo*ΔNterm, UAS-*dNedd4Lo*ΔMid and UAS-*dNedd4Lo* C->A crossed with *24B*-GAL4 or *5*-GAL4 muscle driver at 25°C, RT or 18°C did not result in any lethality (Table S3). Next, we scored for muscle innervation defects from UAS-dNedd4LoΔNterm and UAS-dNedd4LoΔMid transgenic flies as described above for UAS-dNedd4Lo WT and UAS-dNedd4S WT. We found that while both UAS-*dNedd4Lo*ΔMid and UAS-*dNedd4Lo*ΔNterm still exhibited abnormalities compared to the control (*5*-GAL4 muscle driver fly alone), these abnormalities were seen at a much lower frequency than UAS-*dNedd4Lo* WT (Fig. 4C). Furthermore, there was no significant difference between UAS-*dNedd4Lo*ΔMid and UAS-*dNedd4Lo*ΔNterm (p<0.3729). These results show that removing either the N terminal or middle unique regions of dNedd4Lo reduces the frequency of abnormal motor neuron innervation on muscles 13->12 that was observed by muscle-specific overexpression of UAS-*dNedd4Lo* WT. This suggests that the N-terminal and middle unique regions contribute to the innervation defects caused by dNedd4Lo. Furthermore, mutating the catalytic Cys of dNedd4Lo (UAS-*dNedd4Lo* C->A mutant) abolishes the abnormality in muscle innervation, indicating that the catalytic activity of dNedd4Lo is required for its adverse effect on NM synaptogenesis (Fig. 4C). Our previous work showed that overexpressing the catalytic inactive mutant of dNedd4S, dNedd4S C->A, caused significant abnormal muscle innervation (which was not seen upon overexpression of dNedd4S WT [19]). Interestingly, the results we show here for dNedd4Lo are opposite to the results we obtained previously for dNedd4S. Thus, these combined data suggest that while dNedd4S has a positive role in neuromuscular synaptogenesis, dNedd4Lo plays a negative role in this process.

### The negative role of dNedd4Lo in NM synaptogenesis is not caused by its inhibition of catalytic activity of dNedd4S, nor by interfering with dNedd4S-mediated regulation of Comm

**Figure 5:**
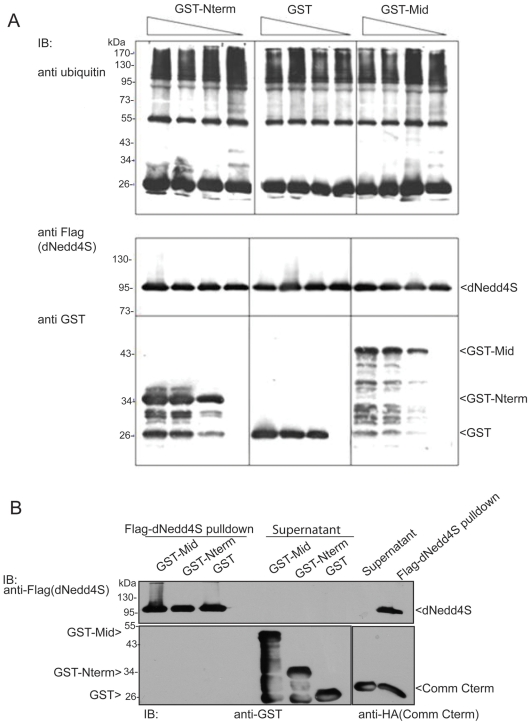
The unique regions of dNedd4Lo do not bind to dNedd4S nor inhibit its catalytic activity. (A) *In vitro* ubiquitylation activity of dNedd4S in the presence of GST alone (control) or GST-tagged dNedd4Lo Nterm or Mid unique regions, detected using anti-ubiquitin antibody on western blots. E1, E2 (UbcH5), E3 (dNedd4S), ubiquitin and ATP were included in the ubiquitylation reactions, as well as increasing concentrations (0.9 µM, 1.8 µM and 3.6 µM) of each potential inhibitor (GST, GST-Nterm, GST-Mid). The blot was stripped and re-blotted with anti-Flag antibody to show equal amount of Flag-dNedd4S (0.27 µM) present in all lanes (upper panel of lower blot), and also re-blotted with anti-GST antibody (lower panel). (B) Lack of *in vitro* binding of dNedd4S to GST fused dNedd4Lo-Nterm, dNedd4Lo-Mid or GST alone. Flag-tagged dNedd4S expressed in HEK293T cells was precipitated from transfected cells, and immunoblotted for Flag (dNedd4, upper panel) or GST (lower panel). HA-tagged C terminus of Comm (which contains PY motifs) was used as a positive control for the binding to dNedd4S (right side of the blot).

To explain the negative role of dNedd4Lo and positive role of dNedd4S in NM synaptogenesis, as well as if/how they co-ordinately regulate this process, we investigated the possibility that dNedd4Lo inhibits the function of dNedd4S by interfering with its catalytic activity. Since the N-terminal and middle unique regions of dNedd4Lo appear to be responsible for its negative effect on fly viability and NM synaptogenesis, we investigated the possibility that they inhibit the catalytic activity of dNedd4S. This was tested in an *in vitro* ubiquitylation and binding assays. Our results show that adding recombinant proteins corresponding to the Nterm or Mid unique regions of dNedd4Lo into the reaction mixture, either alone or together, did not affect ubiquitylation activity of dNedd4S (Fig. 5A and Fig. S2), nor did these proteins directly bind to dNedd4S (Fig. 5B). In contrast, the C-terminal region of Comm, containing the PY motifs, was able to bind well to dNedd4S (as we previously demonstrated, [19]) and hence was used as a positive control for the binding (Fig. 5B).

**Figure 6:**
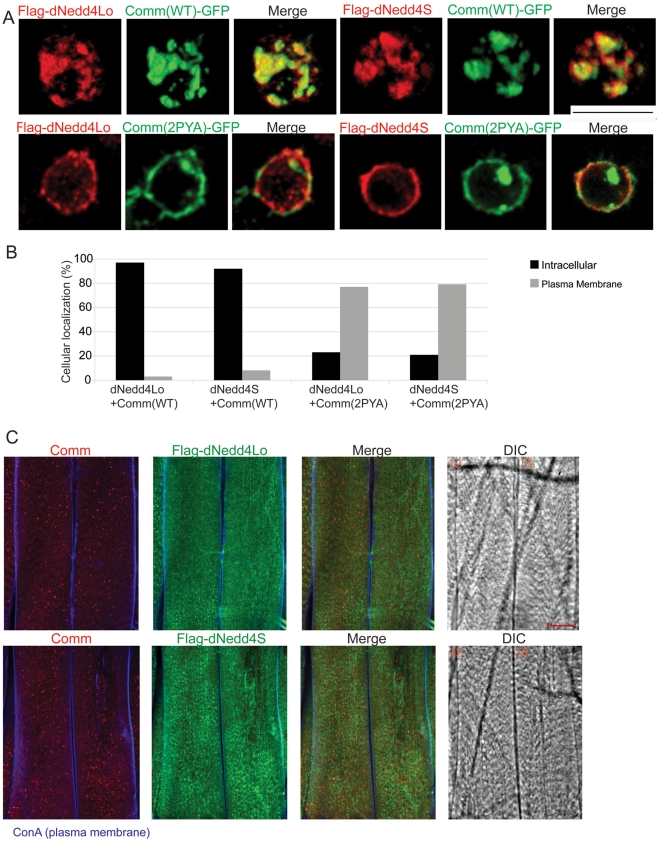
Cellular localization of Comm in S2 cells and muscles in the presence of overexpressed dNedd4Lo. (A,B) Comm-GFP (WT or 2PYA mutant, green) was co-expressed with Flag-dNedd4Lo or Flag-dNedd4S (red) in *Drosophila* S2 cells and their sub-cellular localization (plasma membrane (PM) or intracellular) analyzed by immunofluorescence (A) and quantified (B). In (B), *P*-values were determined with Fisher's exact test (two tailed) on 100 cells per treatment, and show no statistical difference in Comm localization in the presence of overexpressed dNedd4S or dNedd4Lo (dNedd4Lo+Comm WT vs. dNedd4S+Comm WT, P<0.2134; dNedd4Lo+Comm 2PYA vs. dNedd4S+Comm 2PYA, P<0.8646). Scale bar in A, 10 µm. (C) Comm and dNedd4 localization in the muscles (muscles 12 and 13 are shown) of third instar larvae overexpressing Flag-tagged dNedd4Lo (UAS-*dNedd4Lo*/*5*-GAL4) or dNedd4S (UAS-*dNedd4S*/*5*-GAL4), which was stained with anti Comm antibodies (red) or anti Flag (dNedd4) antibodies (green). The plasma membrane of the muscles was stained with ConA (blue). 40–50 larvae (per genotype) were analyzed, and 100% showed the same localization pattern depicted in panel C. Scale bar in panel C, 30 µm.

Another possible way in which dNedd4Lo could negatively regulate NM synaptogenesis is through the regulation of dNedd4S substrates. Since Comm is a known target of dNedd4S in NM synaptogenesis and removal of Comm from the muscle surface is required for initiation of this event [19], the effect of dNedd4Lo overexpression on endocytosis of Comm from the cell surface was studied in *Drosophila* S2 cells, which endogenously express dNedd4S. Comm-GFP (WT or 2PY->A mutant, which cannot bind dNedd4) was co-expressed with Flag-dNedd4Lo or Flag-dNedd4S. We hypothesized that overexpression of dNedd4Lo WT would interfere with the dNedd4S-mediated endocytosis of Comm, if indeed dNedd4Lo were to oppose the function of dNedd4S towards Comm. Surprisingly, we found that when co-expressed with dNedd4Lo or dNedd4S, Comm was properly internalized from the cell surface in most cells and co-localized with dNedd4Lo or dNedd4S in intracellular vesicles. As controls, we showed that Comm 2PY->A mutant remained on the plasma membrane of most cells when co-expressed with either dNedd4S or dNedd4Lo (Fig. 6A,B). Likewise, staining for Comm and dNedd4 in muscles of third instar larvae revealed proper internalization of Comm from the muscle surface in muscles that overexpress dNedd4Lo, much like those overexpressing dNedd4S (Fig. 6C). Thus, the negative effect of dNedd4Lo on NM synaptogenesis was likely not caused by interference with Comm endocytosis, since dNedd4Lo was able to promote Comm internalization similar to dNedd4S.

### Expression of dNedd4Lo is suppressed during NM synaptogenesis

**Figure 7:**
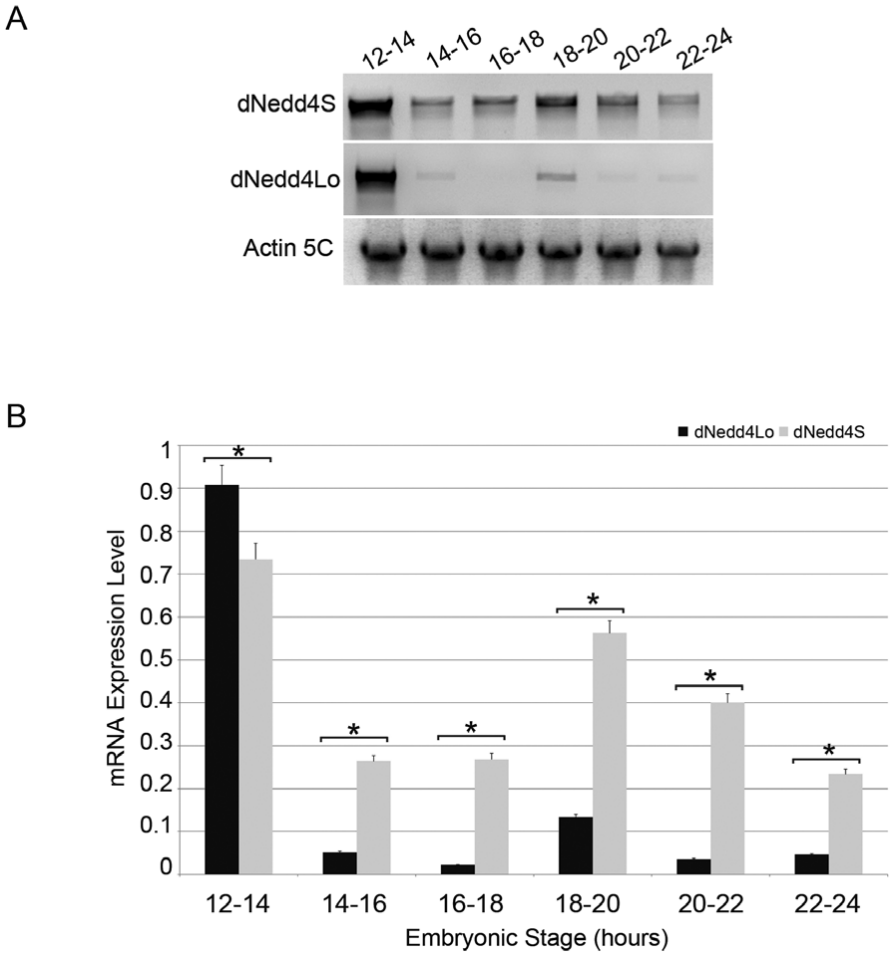
Different expression levels of dNedd4Lo versus dNedd4S during 24 hr embryonic development. (A) Expression of dNedd4S and dNedd4Lo mRNAs were analyzed by semi-quantitative RT-PCR at the indicated embryonic stages. Actin 5c was included as an internal control. (B) The bar chart represents mean expression level ± SD from 4 separate experiments. *indicates a significant difference between dNedd4S and dNedd4Lo (*p*<0.01, Student's *t*-test).

Given the adverse effect of dNedd4Lo on NM synaptogenesis and larval locomotion, in contrast to dNedd4S (which positively regulates NM Synaptogenesis), it is expected that their timing of expression be tightly regulated. Indeed, our semi-quantitative RT-PCR revealed that expression of dNedd4Lo in the embryo is down-regulated soon after the onset of NM synaptogenesis (13 hrs after egg laying), while expression of dNedd4S remains high (Fig. 7). These results suggest that the negative function of dNedd4Lo has to be suppressed during NM synaptogenesis, and that the constant overexpression of UAS-*dNedd4Lo* driven by the GAL4 muscle drivers throughout embryogenesis could have contributed to the observed lethality and muscle innervation defects, as well as abnormal locomotor activity.

## Discussion

We previously showed that dNedd4S is involved in NM synaptogenesis in *Drosophila* [19], most likely by promoting internalization of Comm from the muscle cell surface (a step necessary for initiation of NM synaptogenesis, [23]). Consistent with this model, knock down of dNedd4 during early muscle development or overexpression of Comm mutants that cannot bind dNedd4 yielded the same defects in NM synaptogenesis [19]. Here, we provide genetic evidence that, in contrast to dNedd4S, the splice isoform dNedd4Lo has a negative role in NM synaptogenesis and embryo development in flies. In accord, expression of dNedd4Lo is reduced (and that of dNedd4S is increased) during synaptogenesis, to permit synaptogenesis to proceed. This negative role of dNedd4Lo does not involve Comm or phosphorylation of dNedd4Lo by Akt, nor does it involve an adverse effect of the unique regions of dNedd4Lo on the catalytic activity of the Hect domain of dNedd4S. Instead, it is likely that the unique Nterm and Mid regions of dNedd4Lo contribute to inhibition of NM synaptogenesis by interacting with other cellular factors or complexes, which are not yet known. Studies to analyze differences in general pattern of ubiquitylation upon overexpression of dNedd4Lo vs. dNedd4S in S2 cells did not reveal overt differences (Fig. S3), most likely due to insufficient sensitivity of the system to detect changes in ubiquitylation of specific substrates among the many ubiquitylated cellular proteins.

Our studies here demonstrate that muscle-specific overexpression of dNedd4Lo causes abnormal motor neuron innervation along the SNb branch on body wall muscles 13->12. The types of defects we found include inappropriate backward innervation from muscles 12->13 and increased number of nerve branches on muscle 12. The backward innervation defect was previously observed for overexpression of Comm 2PY->A (that cannot bind dNedd4) and Comm 10K->R mutants (that cannot become ubiquitylated), as well as *dNedd4* RNAi mutants [19]. The other common defect we observed was increased motor nerve branching on muscle 12.

It is known that disruption of genes involved in cell adhesion processes cause nerve branching defects, such as position specific (PS) β-integrin, fasciclinII (FasII), Calcium/Calmodulin dependent Kinase II (CaMKII), and DLG (a PDZ-domain Scaffold protein) [36], [37]. They form a post-synaptic complex in the muscle to co-ordinately regulate defasiculation of the nerve terminal endings and fine-tune the interaction between motor neurons and their muscle targets. It has been proposed that β-integrin regulates recruitment of the cell adhesion molecule FasII on the muscle surface [36]. Down-regulation of β-integrin and up-regulation of FasII on the muscle surface lead to nerve defasiculation. Whether or not the adverse effects of dNedd4Lo on NM synaptogenesis involve these (or other) proteins is currently unknown.

One consequence of muscle innervation defects could be abnormal locomotor activity. Indeed, we found a significant reduction in the locomotor activity of larvae that overexpress dNedd4Lo specifically in the muscle. Furthermore, muscle-specific overexpression of dNedd4Lo leads to lethality during development. However, the muscle drivers 24B-GAL4 and 5-GAL4, used in this experiment, drive expression in the entire mesoderm [29], [32], which derives into somatic (body wall) muscles for movement [33], [34], visceral (gut) muscles for digestion, and cardiac muscles [35]. Thus, while the innervation defects on body wall muscles may contribute to the larval lethality, defects in heart and/or gut muscle functions might contribute as well, since heart activity and feeding are essential for larval survival.

Similar muscle innervation defects were also observed for the mouse homologue of dNedd4, mNedd4 (mNedd4-1) [20]. In *mNedd4* mutant embryos, motor nerves defasciculate upon reaching their skeletal muscle targets and the pre-synaptic nerve terminal branches are increased in number. It was also demonstrated that *mNedd4* mutants had increased spontaneous miniature endplate potential (mEPP) frequency, which is consistent with the ultra structural alternation. In addition, β-catenin, a subunit of the cadherin protein complex, was proposed to be a potential substrate for mNedd4 in NM synapse formation and function. β-catenin deficient muscles show similar defects of nerve defasiculation as *mNedd4* mutant [38]. Similarly, molecular manipulation of β-integrins, which is also involved in the cell adhesion process, in muscles of mice also lead to abnormal development of pre-synaptic nerve terminals [39].

Phosphorylation is an important mechanism for the regulation of Nedd4 proteins and other E3 ubiquitin ligases. For example, Nedd4-2 is known to be regulated by Akt/Sgk – mediated phosphorylation, which inhibits its ability to interact with its substrate ENaC [13], [14]. However, mutating the dAkt phosphorylation sites (S->A) in dNedd4Lo did not affect the abnormal muscle innervation. Since the dAkt phosphorylation site in the unique N-terminal region of dNedd4Lo did not explain its negative function, we removed the whole unique N-terminal region or the middle region to determine their role in the negative effect of dNedd4Lo on viability and NM synaptogenesis. We demonstrated that removing either the N-terminal or the middle region rescued the lethality and alleviated the muscle innervation defects. Therefore, both regions are involved in the negative function of dNedd4Lo in this event. We thus investigated two possible underlying mechanisms for the negative regulation of dNedd4Lo in NM synaptogenesis. First, we tested inhibition of function of dNedd4S through the unique regions of dNedd4Lo. It is known that the catalytic activity of Nedd4 proteins can be regulated through auto-inhibition mechanism. For example, the WW domains of Nedd4-2 [40] and a close relative of Nedd4, Itch [41], as well as the C2 domain of Smurf 2 [42], were shown to bind to their own Hect domains and inhibit their catalytic activity. However, our data suggest that the unique N-terminal or middle regions of dNedd4Lo do not bind nor inhibit the catalytic activity of dNedd4S *in vitro*. Second, we investigated the effect of dNedd4Lo overexpression on dNedd4S-mediated Comm endocytosis in *Drosophila* S2 cells and body wall muscles. We hypothesized that if dNedd4Lo acts to inhibit the function of dNedd4S, it would interfere with Comm endocytosis. However, our results show that overexpression of dNedd4Lo did not affect internalization of Comm. Therefore, the unique regions of dNedd4Lo regulate NM synaptogenesis by as yet unknown mechanisms, possibly by targeting other substrates.

Interestingly, differential regulation of substrates is known for isoforms of the E3 ligase Cbl, namely dCblL (long) and dCblS (short). While the long isoform down-regulates EGFR signaling, the short isoform preferentially controls Notch signaling through regulation of the Notch ligand Delta [43]. DNedd4 might use a similar mechanism to regulate *Drosophila* embryo development, particularly NM synaptogenesis. In addition, temporal regulation of expression of dNedd4S and dNedd4Lo differs, allowing NM synaptogenesis to proceed at the appropriate time in development.

## Materials and Methods

### Constructs

#### Generation of UAS-*dNedd4S* (WT), UAS-*dNedd4Lo* (WT), UAS-*dNedd4S* (S444->A), and UAS-*dNedd4Lo* (S39->A, S645->A) constructs

*dNedd4Lo* cDNA was kindly provided by Dr. Guy Tear. PCR-amplified full-length *dNedd4S* (S444->A) and *dNedd4Lo* (S39->A, S645->A) mutants were synthesized using the Quick Change Site Directed Mutagenesis Kit (Stratagene). The constructs were subcloned into pUAST, KpnI & BamHI sites with an N-terminal Flag tag to generate transgenic lines, as described [29].

#### Generation of UAS-*dNedd4Lo*ΔNterm, UAS-*dNedd4Lo*ΔMid, and UAS-*dNedd4Lo* C->A mutants

UAS-*dNedd4Lo*ΔNterm: The DNA fragment flanked by the AarI & Bsp1407I sites of Flag-*dNedd4Lo* WT in pRmHa3 vector was subcloned to replace the region in the Flag-*dNedd4S* WT-pRmHa3 flanked by the same restriction sites. UAS-*dNedd4Lo*ΔMid: the DNA fragment flanked by the AarI&Bsp1407I sites of Flag-*dNedd4S* WT in pRmHa3 vector was subcloned to replace the region in the Flag-*dNedd4Lo* WT-pRmHa3 flanked by the same restriction sites.

UAS-*dNedd4Lo* C->A: the DNA fragment flanked by the XhoI & BamHI sites containing the Cys->Ala mutation (generated by site-directed mutagenesis) of Flag-*dNedd4S* C->A in pRmHa3 vector was subcloned to replace the region in the Flag-*dNedd4Lo* WT-pRmHa3 flanked by the same restriction sites. The constructs were subsequently subcloned into pUAST KpnI & BamHI sites to generate transgenic lines, as described [29].

#### Generation of GST-dNedd4Lo N-terminus (Nterm) and GST-dNedd4L Middle (Mid) for bacterial expression and purification

The PCR-amplified DNA fragment corresponding to the N-terminal unique sequence of *dNedd4Lo* (bp 1 to 189) was subcloned into pGEX6P1 BamHI & SalI sites with an N-terminal GST tag. The PCR-amplified DNA fragment corresponding to the middle unique sequence of *dNedd4Lo* (bp 912 to 1419) was subcloned into pGEX6P1 BamHI & EcoRI with an N-terminal GST tag. All fly crosses are summarized in Table S4.

### Rescue of dNedd4 null mutant flies

The fly lines and final crosses generated to rescue *dNedd4^T121FS^* homozygote mutant flies with UAS-*dNedd4Lo* WT or UAS-*dNedd4S* WT are listed in the Table S4. Adult flies in final crosses were put on grape agar plates to lay eggs, which were collected and transferred to new plates after ∼16 hrs. Plates were observed under a fluorescent microscope to look for rescued larvae, which did not express GFP (non-GFP).

### Semi-quantitative RT-PCR

*Oregon-R* wild-type flies were put on fruit agar plates to lay eggs, which were collected every 2 hours during the 24-hr embryonic development at ∼22°C. Embryos were incubated in 50% bleach for 5 min, collected using embryo collection net (Netwell insert, *Corning*, Acton, MA), and rinsed in PBS (phosphate-buffered saline). Total RNA was extracted from embryos (Qiagen RNeasy Mini Kit) and reverse transcribed to cDNA (Invitrogen SuperScript III Synthesis System). Forward primer: ccatggctgcaataacagtg and reverse primer: gcgtagttcgcgtgttatga were used to amplify dNedd4S to give a PCR product of 1124 bp using Platinum Taq DNA Polymerase (Invitrogen). Forward primer: tacactcctcgcagatcgtt and reverse primer: gtgtctctgaccccgatgtt were used to amplify dNedd4Lo to give a PCR product of 1239 bp. For Actin 5C: forward primer: tgtgtgacgaagaagttgctg, and reverse primer: cctcctccagcagaatcaag were used to amplify this internal control, yielding a PCR product of 1193 bp. 50 ng of each cDNA sample was added to a 50 µl PCR mixture. PCR conditions were as follows: 94°C for 30 sec, 55°C for 30 sec, and 68°C for 2 min. A series of amplification cycles from 28 to 42 were tested to determine the exponential phase and 34-cycle was chosen to perform the PCR. Equal volumes of the resulting PCR reactions were analyzed by electrophoresis on a 1.5% agarose gel. Band intensities were measured using Image J software to quantify the expression level of each mRNA at each given embryonic stage.

### Biochemical assays

For expression of UAS-*dNedd4S* (WT and mutants) and UAS-*dNedd4Lo* (WT and mutants) lines. The UAS-*dNedd4S* (WT and S444->A [S->A]) or UAS-*dNedd4Lo* (WT, S39->A,S645->A), ΔNterm, ΔMid, C->A)×*daughterless(da)*-GAL4 embryos were collected at room temperature (RT). The embryos were dechorionated with 50% bleach for 5 min and lysed with lysis buffer (150 mM NaCl, 50 mM HEPES, 1% Triton-X, 10% glycerol, 1.5 mM MgCl_2_ and 1.0 mM EGTA) plus protease inhibitors (1 mM PMSF, 1 µg/ml each of aprotinin, leupeptin and pepstatin A) to examine expression by Western blotting.

Antibodies used for this and all other experiments are summarized in Table S5.

### Phosphorylation assays

S2 cells were transiently transfected with Flag-tagged dNedd4S [WT, dNedd4S(S444A)], or dNedd4Lo [WT,dNedd4Lo(S39A), dNedd4Lo(S39A,S645A)] in the pRmHA3 vector and lysed in lysis buffer containing phosphatase inhibitors (10 mM NaF, 10 mM Na pyrophosphate, 10 mM Na-orthovanadate). DNedd4S (WT or mutants) or dNedd4Lo (WT or mutants) was immunoprecipitated (IP) with anti-Flag antibodies and blotted with anti Phospho-(Ser/Thr) Akt substrate antibodies (1∶1000,Cell Signaling Technology).

### Larva dissections and immunostaining

Pre-wandering third instar larvae were dissected using a standard fillet preparation technique [44], fixed in 4% paraformaldehyde (PFA) (20 min), washed in PBT (0.1% Tween-20 in PBS), blocked, and stained with Cy3-conjugated anti-horseradish peroxidase (HRP) antibody to visualize motor neurons, and Alexa Fluor^488^ Phalloidin to visualize body wall muscles. Epifluorescence microscopy and imaging of the neurons and muscles was performed using an LSM 510 confocal microscope.

### S2 cells immunostaining

Live S2 cells co-expressing Comm (WT or 2PY->A)-GFP [19] and Flag-dNedd4 (S or Lo, WT or C->A) were washed, fixed in 2% PFA (10 min), permeabilized with 0.1% Saponin, stained with M2 anti-Flag antibody and Cy3-conjugated goat anti-mouse antibody (Table S5), and visualized by confocal microscopy.

### *In vitro* ubiquitylation assays

Full-length dNedd4S protein immunopurified from HEK293T cells was incubated in reaction mixtures containing 100 nM human E1 ubiquitin-activating enzyme (BostonBiochem), 200 nM human E2 ubiquitin-conjugating enzyme (UbcH5; BostonBiochem), 1 µg of ubiquitin (Sigma) and 4 mM ATP in a reaction buffer (25 mM Tris/HCl pH 7.5, 50 mM NaCl, 0.1 mM dithiothreitol and 4 mM MgCl_2_). Reactions were incubated for 1 hr at RT. Protein ubiquitylation was detected on western blots. Membranes were blocked and incubated with mouse anti-ubiquitin antibody and HRP-conjugated goat-anti-mouse antibody to detect ubiquitin (Table S5). To detect Flag-dNedd4S, membranes were incubated with M2 anti-Flag antibody and HRP-conjugated goat anti-mouse antibody. Mouse anti-glutathione S transferase (GST) antibody and HRP-conjugated goat anti-mouse antibody were used to detect bacterially expressed GST-dNedd4Lo Nterm and GST-dNedd4Lo Mid.

### *In vitro* binding assay

Flag-dNedd4S protein immunopurified from HEK293T cells was immobilized on M2 anti-Flag agarose beads (Sigma) and incubated with bacterially expressed GST-dNedd4Lo Nterm or GST-dNedd4Lo Mid at 4°C for 2 hrs. Supernatant was removed and the beads were washed with lysis buffer. Mouse anti-GST antibody and HRP-conjugated goat-anti-mouse antibody were used to detect GST-dNedd4Lo Nterm or GST-dNedd4Lo Mid (Table S5). Flag-dNedd4S was detected using M2 anti-Flag antibody and HRP-conjugated goat anti-mouse antibody.

### Larval Locomotor Activity

Fly crosses generated to analyze larval locomotion are listed in Table S4. 150-sec path length was determined for the following heterozygous genotypes: (1) Driver control line, +/*24B*-GAL4; (2) UAS control line, +/UAS-*dNedd4Lo*; and (3) Over-expression line, +/UAS-*dNedd4Lo*; +/*24B*-GAL4 to analyze their locomotor activity. All experiments were performed at 24±1°C in a climate-controlled room with 45–50% humidity with a 12/12-hr light/dark cycle. All animals were tested at the pre-wandering physiological stage and the stage was confirmed as previously described [45]. The same number of 5 day old adult flies were put on grape agar plates to lay eggs and transferred to new plates every 24 hrs. After 28 hrs, hatched larvae were cleared from the plate and all newly hatched larvae were collected 2 hrs later. Twenty larvae (3 repetitions per genotype) were separated into vials containing standard fly food medium. Larvae from over-expression lines showed a delay in development, taking 172±4 h after egg-laying to reach the wandering stage, while control larvae reached this stage after 164±4 h. Thus, for all experiments, larvae were aged 168±2 h for the over-expression line and 158±2 h for controls. To measure locomotor activity, larvae were rinsed and placed in a Petri dish containing 3% grape agarose for 30 sec to adapt to the experimental conditions. The crawling path for each larva was traced for 150 sec on the lid of the dish and scanned for subsequent digital analysis. Image J software (National Institutes of Health) was used for quantification of crawling path length.

### Statistical Analyses

GraphPad Prism 5.0 software (GraphPad Software, San Diego, CA) and Fisher's exact test in a 2×2 contingency table were used to analyze the data of motor neuron innervation on body wall muscles, as well as protein localization of dNedd4 and Comm in S2 cells. A p-value of <0.05 was considered significant. For analysis of locomotor activity, the behavioral data were Box-Cox transformed to correct problems of distribution and non-homogeneity of variance before transformation. Normal distribution was confirmed by the Kolmogorov-Smirnov test. The common variance of the three lines was tested by the Levene test (F_2, 168_ = 2.47, p = 0.087). One-way ANOVA and subsequent Turkey post-hoc comparisons were performed to evaluate differences among lines. A p-value of <0.01 was considered significant. Statistical analyses were conducted using Statistica 8.0 (StatSoft) software. For analysis using semi-quantitative RT-PCR, Student's *t*-test was used to compare the mRNA expression level of dNedd4Lo versus dNedd4S at each given developmental stage during embryogenesis. A p-value of <0.01 was considered significant.

## Supporting Information

Figure S1 **Catalytic activity of dNedd4S and dNedd4Lo, and normal cellular localization of these isoforms ectopically expressed in salivary glands of third instar larvae.** (A) Catalytic activity of dNedd4S and dNedd4Lo: *In vitro* ubiquitylation assay of wildtype (WT) dNedd4S or dNedd4Lo was performed by incubating E1, E2 (UbcH5), E3 (Flag-tagged dNedd4S or dNedd4Lo), ubiquitin and ATP, and the extent of ubiquitylation (most likely reflecting autoubiquitylation) analyzed by immunoblotting with anti-ubiquitin antibodies. Note the loss of ubiquitylation of the catalytically-inactive C->A mutants of the dNedd4 isoforms. Lower panels: The blot was stripped and re-blotted with anti-Flag antibody to show equal amounts of Flag-dNedd4S WT and its C->A mutant (left blot) or Flag-dNedd4Lo WT and C->A mutant (right blot) present in the reactions. (B) Similar to dNedd4S, FLAG-dNedd4Lo WT and its S->A mutant do not form aggregates and localize ubiquitously in the cytosol and on the plasma membrane, but not in the nucleus (stained with DraQ5 in blue). W^1118^ fly was used as a negative control to show no background staining of the anti-FLAG antibody (green). Scale bars, 10 µm.
(TIF)

Figure S2 **The unique regions of dNedd4Lo do not inhibit catalytic activity of dNedd4S.** *In vitro* ubiquitylation activity of dNedd4S in the presence of GST alone (control), GST-tagged dNedd4Lo Nterm, Mid, or both unique regions, detected using anti-ubiquitin antibody on western blots. E1, E2 (UbcH5), E3 (dNedd4S), ubiquitin and ATP were included in the ubiquitylation reactions, as well as increasing concentrations (0.9 µM, 1.8 µM and 3.6 µM) of each potential inhibitor (GST, GST-Nterm, GST-Mid, or GST-Nterm+GST-Mid). Middle panel: The blot was stripped and re-blotted with anti-Flag antibody to show equal amount of Flag-dNedd4S present in all lanes. Bottom panel: The blot was also stripped and re-blotted with anti-GST antibody. The catalytically inactive dNedd4S C->A mutant was included as a negative control to demonstrate that the ubiquitylation activity observed was mediated by dNedd4S.
(TIF)

Figure S3 **Ubiquitylation of cellular proteins in S2 cells ectopically overexpressing dNedd4Lo or dNedd4S.** S2 cells were untransfected or transfected with Flag-tagged dNedd4S (WT or its catalytically-inactive CA mutant) or dNedd4Lo (WT or its catalytically-inactive CA mutant), and extent/pattern of ubiquitylation of cellular proteins analyzed by immunoblotting (IB) with anti ubiquitin antibodies (top panel). Bottom panels depict controls for dNedd4Lo and dNedd4S expression and for loading controls (lamin). In the bottom panels, double the amount of proteins were loaded on the gel as compared with the respective top (ubiquitylation) panel.
(TIF)

Table S1 **Lethality test for ubiquitous overexpression of dNedd4S (WT and S->A mutant) and dNedd4Lo (WT and S->A mutant) using different GAL4 enhancer drivers at different temperatures.**
(DOCX)

Table S2 **Lethality test for overexpression of dNedd4S WT, dNedd4Lo WT and their S->A mutants in different tissues using tissue-specific GAL4 enhancer drivers.**
(DOCX)

Table S3 **Lethality test for ubiquitous (Daughterless, Actin, and Tubulin) and muscle-specific (24B and 5) overexpression of UAS-** ***dNedd4Lo*** **ΔNterm, UAS-** ***dNedd4Lo*** **ΔMid and UAS-** ***dNedd4Lo*** **C->A using different GAL4 enhancer drivers at different temperatures.**
(DOCX)

Table S4 **Fly line crosses used for the experiments.**
(DOCX)

Table S5 **Antibodies used for staining.**
(DOCX)
