# Supplementary figures and images for: A Splice Isoform of DNedd4, DNedd4-Long, Negatively Regulates Neuromuscular Synaptogenesis and Viability in Drosophila
Source: PLoS One. 2011 Nov 14;6(11):e27007. doi: 10.1371/journal.pone.0027007 (PMC3215714; doi:10.1371/journal.pone.0027007)

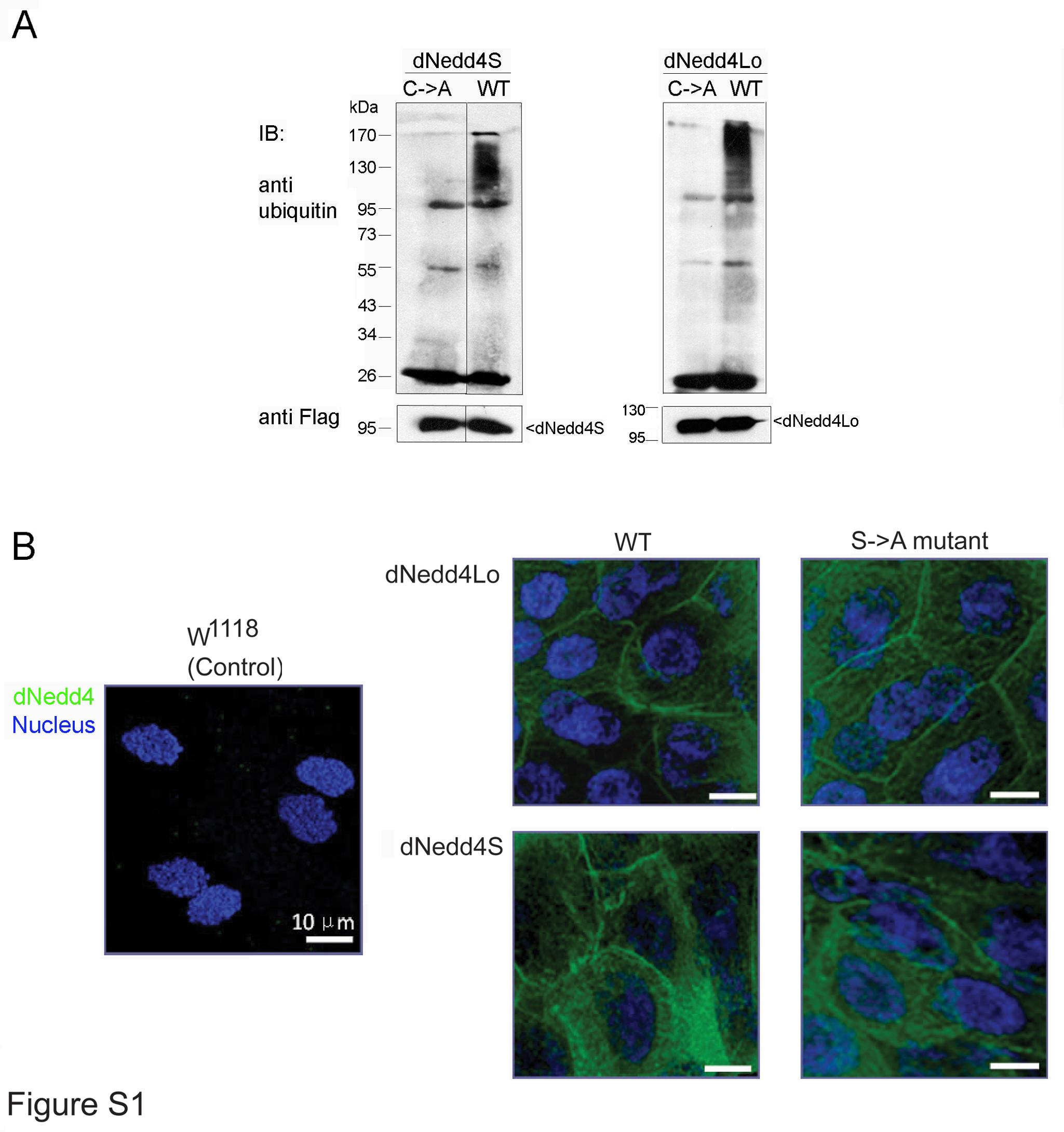

Supplement: Figure S1 — Catalytic activity of dNedd4S and dNedd4Lo, and normal cellular localization of these isoforms ectopically expressed in salivary glands of third instar larvae. (A) Catalytic activity of dNedd4S and dNedd4Lo: In vitro ubiquitylation assay of wildtype (WT) dNedd4S or dNedd4Lo was performed by incubating E1, E2 (UbcH5), E3 (Flag-tagged dNedd4S or dNedd4Lo), ubiquitin and ATP, and the extent of ubiquitylation (most likely reflecting autoubiquitylation) analyzed by immunoblotting with anti-ubiquitin antibodies. Note the loss of ubiquitylation of the catalytically-inactive C->A mutants of the dNedd4 isoforms. Lower panels: The blot was stripped and re-blotted with anti-Flag antibody to show equal amounts of Flag-dNedd4S WT and its C->A mutant (left blot) or Flag-dNedd4Lo WT and C->A mutant (right blot) present in the reactions. (B) Similar to dNedd4S, FLAG-dNedd4Lo WT and its S->A mutant do not form aggregates and localize ubiquitously in the cytosol and on the plasma membrane, but not in the nucleus (stained with DraQ5 in blue). W1118 fly was used as a negative control to show no background staining of the anti-FLAG antibody (green). Scale bars, 10 µm. (TIF) [file pone.0027007.s001.tif]

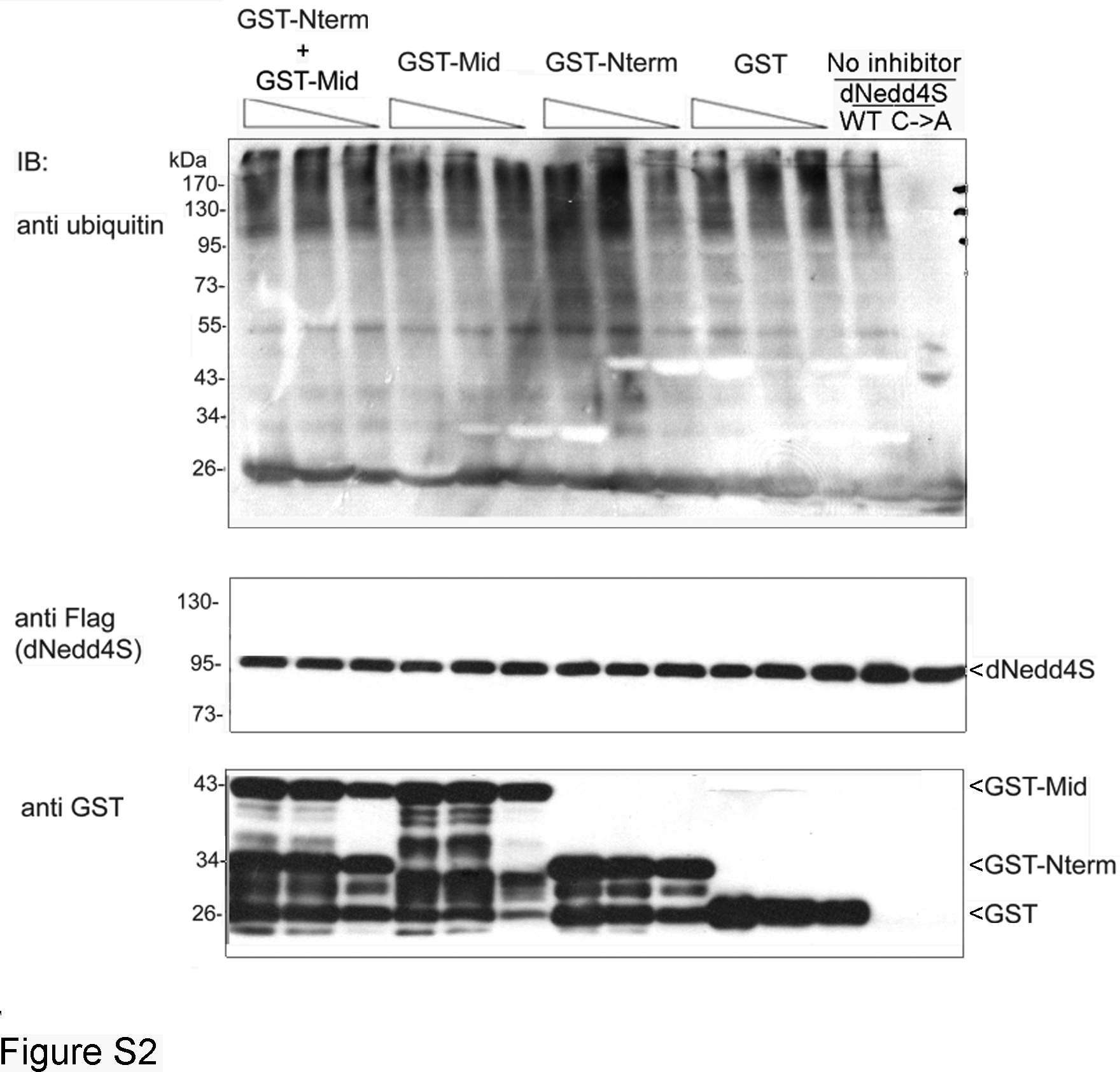

Supplement: Figure S2 — The unique regions of dNedd4Lo do not inhibit catalytic activity of dNedd4S. In vitro ubiquitylation activity of dNedd4S in the presence of GST alone (control), GST-tagged dNedd4Lo Nterm, Mid, or both unique regions, detected using anti-ubiquitin antibody on western blots. E1, E2 (UbcH5), E3 (dNedd4S), ubiquitin and ATP were included in the ubiquitylation reactions, as well as increasing concentrations (0.9 µM, 1.8 µM and 3.6 µM) of each potential inhibitor (GST, GST-Nterm, GST-Mid, or GST-Nterm+GST-Mid). Middle panel: The blot was stripped and re-blotted with anti-Flag antibody to show equal amount of Flag-dNedd4S present in all lanes. Bottom panel: The blot was also stripped and re-blotted with anti-GST antibody. The catalytically inactive dNedd4S C->A mutant was included as a negative control to demonstrate that the ubiquitylation activity observed was mediated by dNedd4S. (TIF) [file pone.0027007.s002.tif]

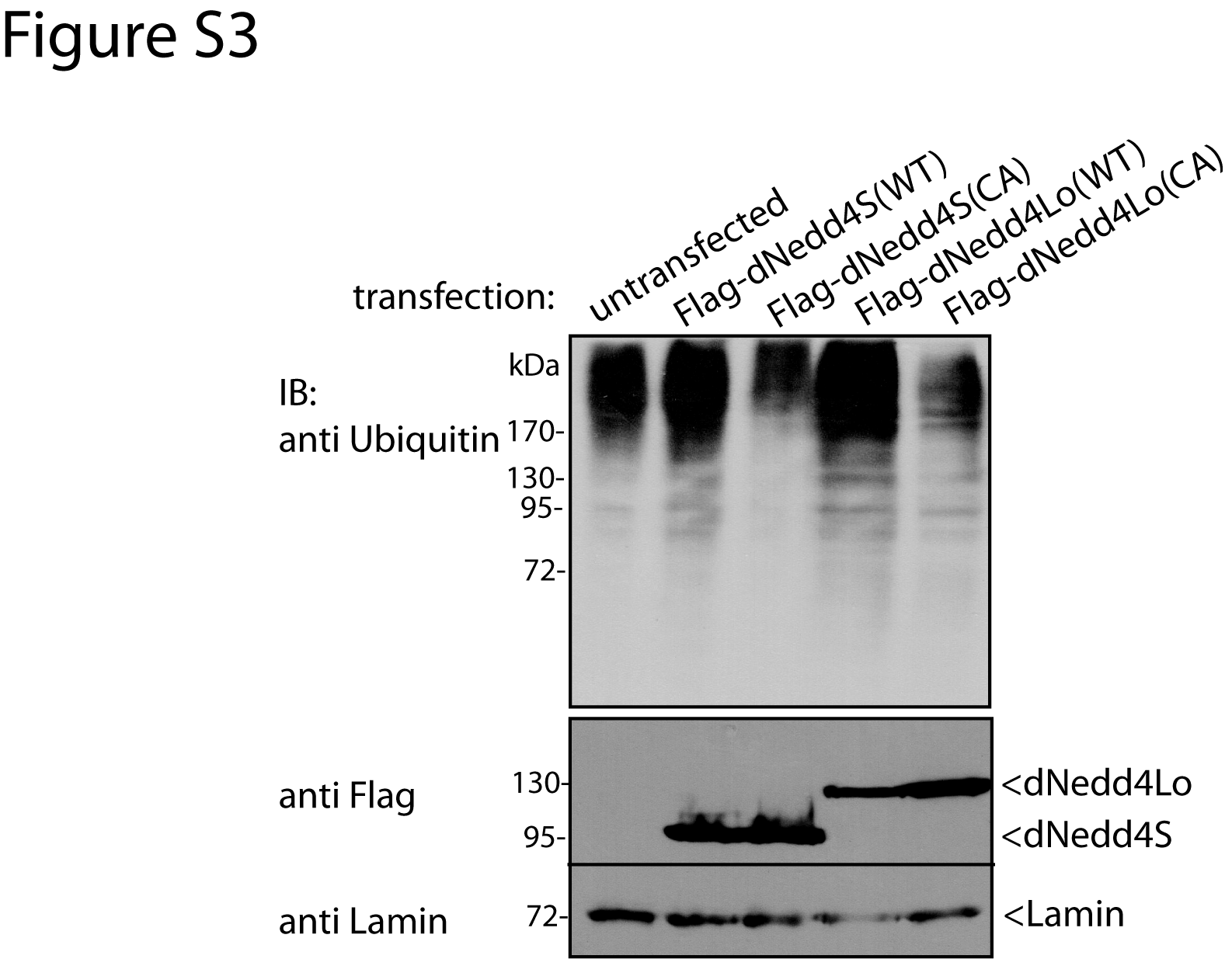

Supplement: Figure S3 — Ubiquitylation of cellular proteins in S2 cells ectopically overexpressing dNedd4Lo or dNedd4S. S2 cells were untransfected or transfected with Flag-tagged dNedd4S (WT or its catalytically-inactive CA mutant) or dNedd4Lo (WT or its catalytically-inactive CA mutant), and extent/pattern of ubiquitylation of cellular proteins analyzed by immunoblotting (IB) with anti ubiquitin antibodies (top panel). Bottom panels depict controls for dNedd4Lo and dNedd4S expression and for loading controls (lamin). In the bottom panels, double the amount of proteins were loaded on the gel as compared with the respective top (ubiquitylation) panel. (TIF) [file pone.0027007.s003.tif]
