# Supplementary material for: A Splice Isoform of DNedd4, DNedd4-Long, Negatively Regulates Neuromuscular Synaptogenesis and Viability in Drosophila
Source: PLoS One. 2011 Nov 14;6(11):e27007. doi: 10.1371/journal.pone.0027007 (PMC3215714; doi:10.1371/journal.pone.0027007)
Supplement: Table S1 — Lethality test for ubiquitous overexpression of dNedd4S (WT and S->A mutant) and dNedd4Lo (WT and S->A mutant) using different GAL4 enhancer drivers at different temperatures. (DOCX) [file pone.0027007.s004.docx]

**Table S1**. Lethality test for ubiquitous overexpression of dNedd4S (WT and S->A mutant) and dNedd4Lo (WT and S->A mutant) using different GAL4 enhancer drivers at different temperatures.

| Ubiquitous GAL4 Driver  UAS  Transgenic Line | | Daughterless (da) | | | Actin | | | Tubulin | | |
| --- | --- | --- | --- | --- | --- | --- | --- | --- | --- | --- |
|  |  | 25^o^C | 22^o^C | 18^o^C | 25^o^C | 22^o^C | 18^o^C | 25^o^C | 22^o^C | 18^o^C |
| dNedd4S | WT |  |  |  |  |  |  |  |  |  |
|  | S->A mutant |  |  |  |  |  |  |  |  |  |
| dNedd4Lo | WT |  |  |  |  |  |  |  |  |  |
|  | S->A mutant |  |  |  |  |  |  |  |  |  |

Note: For each cross, ~30 to 40 progenies were analyzed. α denotes survival to adult stage.

 denotes lethality before adult stage (and stage of lethality varies from embryonic to third instar larval stage).
