# Supplementary material for: A Splice Isoform of DNedd4, DNedd4-Long, Negatively Regulates Neuromuscular Synaptogenesis and Viability in Drosophila
Source: PLoS One. 2011 Nov 14;6(11):e27007. doi: 10.1371/journal.pone.0027007 (PMC3215714; doi:10.1371/journal.pone.0027007)
Supplement: Table S2 — Lethality test for overexpression of dNedd4S WT, dNedd4Lo WT and their S->A mutants in different tissues using tissue-specific GAL4 enhancer drivers. (DOCX) [file pone.0027007.s005.docx]

**Table S2**. Lethality test for overexpression of dNedd4S WT, dNedd4Lo WT and their S->A mutants in different tissues using tissue-specific GAL4 enhancer drivers.

| Tissue-specific  GAL4 Driver  UAS  Transgenic Line | | Mesoderm | | | Ectoderm | | | | Endoderm |
| --- | --- | --- | --- | --- | --- | --- | --- | --- | --- |
|  |  | Muscle | | Fat Body | Motor Neuron | Central Nervous System | Eye | | Respiratory and GI Tract |
|  |  | 24B | 5 | Ppl | D42 | Elav^c155^ | GMR | Ey^129^ | 48Y |
| dNedd4S | WT |  |  |  |  |  |  |  |  |
|  | S->A mutant |  |  |  |  |  |  |  |  |
| dNedd4Lo | WT |  |  |  |  |  |  |  |  |
|  | S->A mutant |  |  |  |  |  |  |  |  |

Note: For each cross, ~30 to 40 progenies were analyzed. Each test was performed at 25^o^C, RT (~22^o^C) and 18^o^C.α denotes survival to adult stage.  denotes lethality before adult stage (and stage of lethality varies from first instar larval stage to early pupal stage).
