# Supplementary material for: A Splice Isoform of DNedd4, DNedd4-Long, Negatively Regulates Neuromuscular Synaptogenesis and Viability in Drosophila
Source: PLoS One. 2011 Nov 14;6(11):e27007. doi: 10.1371/journal.pone.0027007 (PMC3215714; doi:10.1371/journal.pone.0027007)
Supplement: Table S3 — Lethality test for ubiquitous (Daughterless, Actin, and Tubulin) and muscle-specific (24B and 5) overexpression of UAS- dNedd4Lo ΔNterm, UAS- dNedd4Lo ΔMid and UAS- dNedd4Lo C->A using different GAL4 enhancer drivers at different temperatures. (DOCX) [file pone.0027007.s006.docx]

**Table S3**. Lethality test for ubiquitous (Daughterless, Actin, and Tubulin) and muscle-specific (24B and 5) overexpression of UAS-*dNedd4Lo*△Nterm, UAS-*dNedd4Lo*△Mid and UAS-*dNedd4Lo* C->A using different GAL4 enhancer drivers at different temperatures.

| Ubiquitous GAL4 Driver  UAS  Transgenic Line | Daughterless | | | Actin | | | Tubulin | | | 24B | | 5 | |
| --- | --- | --- | --- | --- | --- | --- | --- | --- | --- | --- | --- | --- | --- |
|  | 25^o^C | 22 ^o^C | 18^o^C | 25^o^C | 22 ^o^C | 18^o^C | 25^o^C | 22 ^o^C | 18^o^C | 22^o^C | 25^o^C | 22^o^C | 25^o^C |
| dNedd4Lo△Nterm |  |  |  |  |  |  |  |  |  |  |  |  |  |
| dNedd4Lo△Mid |  |  |  |  |  |  |  |  |  |  |  |  |  |
| dNedd4Lo C->A |  |  |  |  |  |  |  |  |  |  |  |  |  |

Note: For each cross, ~30 to 40 progenies were analyzed. α denotes survival to adult stage.

 denotes lethality before adult stage (and stage of lethality varies from third instar larval stage to late pupal stage).
