# Supplementary material for: A Splice Isoform of DNedd4, DNedd4-Long, Negatively Regulates Neuromuscular Synaptogenesis and Viability in Drosophila
Source: PLoS One. 2011 Nov 14;6(11):e27007. doi: 10.1371/journal.pone.0027007 (PMC3215714; doi:10.1371/journal.pone.0027007)
Supplement: Table S4 — Fly line crosses used for the experiments. (DOCX) [file pone.0027007.s007.docx]

| **Ubiquitous overexpression of dNedd4** |  |  | |
| --- | --- | --- | --- |
| UAS-*dNedd4S WT* | X | *Daughterless*-GAL4 / *Actin*-GAL4 / *Tubulin*-GAL4 [source: Bloomington *Drosophila* Stock Center (Bloomington)] | |
| UAS-*dNedd4S S->A* | X | *Daughterless*-GAL4 / *Actin*-GAL4 / *Tubulin*-GAL4 | |
| UAS-*dNedd4Lo WT* | X | *Daughterless*-GAL4 / *Actin*-GAL4 / *Tubulin*-GAL4 | |
| UAS-*dNedd4Lo S->A* | X | *Daughterless*-GAL4 / *Actin*-GAL4 / *Tubulin*-GAL4 | |
| UAS-*dNedd4Lo△Nterm* | X | *Daughterless*-GAL4 / *Actin*-GAL4 / *Tubulin*-GAL4 | |
| UAS-*dNedd4Lo△Mid* | X | *Daughterless*-GAL4 / *Actin*-GAL4 / *Tubulin*-GAL4 | |
| **Overexpression of dNedd4 in the muscle** | | | |
| *W^1118^* | X | *5*-GAL4 (control) [source: A.Chiba] | |
| *Oregon-R* | X | *24B*-GAL4 (driver control) [source: Bloomington] | |
| *Oregon-R* | X | UAS-*dNedd4Lo WT* (UAS control) | |
| UAS-*dNedd4S WT* | X | *24B*-GAL4 / *5*-GAL4 | |
| UAS-*dNedd4S S->A* | X | *24B*-GAL4 / *5*-GAL4 | |
| UAS-*dNedd4Lo WT* | X | *24B*-GAL4 / *5*-GAL4 | |
| UAS-*dNedd4Lo S->A* | X | *24B*-GAL4 / *5*-GAL4 | |
| UAS-*dNedd4Lo△Nterm* | X | *24B*-GAL4 / *5*-GAL4 | |
| UAS-*dNedd4Lo△Mid* | X | *24B*-GAL4 / *5*-GAL4 | |
| **Overexpression of dNedd4 in various tissues: central nervous system / motor neuron / eye / epithelial lining of digestive system and respiratory system / fat body** | | | |
| UAS-*dNedd4S WT* | X | *elav*^c155^ -GAL4 / *42D-*GAL4 / *GMR*-GAL4 / *ey129*-GAL4 / *48Y*-GAL4 / *Pumpless*-GAL4 [source: G.Boulianne] | |
| UAS-*dNedd4S S->A* | X | *elav*^c155^ -GAL4 / *42D-*GAL4 / *GMR*-GAL4 / *ey129*-GAL4 / *48Y*-GAL4 / *Pumpless*-GAL4 | |
| UAS-*dNedd4Lo WT* | X | *elav*^c155^-GAL4 / *42D-*GAL4 / *GMR*-GAL4 / *ey129*-GAL4 / *48Y*-GAL4 / *Pumpless*-GAL4 | |
| UAS-*dNedd4Lo S->A* | X | *elav*^c155^-GAL4 / *42D-*GAL4 / *GMR*-GAL4 / *ey129*-GAL4 / *48Y*-GAL4 / *Pumpless*-GAL4 | |
| UAS-*dNedd4Lo△Nterm* | X | *elav*^c155^-GAL4 / *42D-*GAL4 / *GMR*-GAL4 / *ey129*-GAL4 / *48Y*-GAL4 / *Pumpless*-GAL4 | |
| UAS-*dNedd4Lo△Mid* | X | *elav*^c155^-GAL4 / *42D-*GAL4 / *GMR*-GAL4 / *ey129*-GAL4 / *48Y*-GAL4 / *Pumpless*-GAL4 | |
| **Rescue of *dNedd4* null flies** | | | |
| +; UAS-*dNedd4S*; *dNedd4*^T121FS^/TM3SerGFP | | X | +; *Actin*-GAL4/CyO, y+; *dNedd4*^T121FS^/TM3SerGFP |
| UAS-*dNedd4Lo*; Sp/CyO,y+; *dNedd4*^T121FS^/TM3SerGFP | | X | +; *Actin*-GAL4/CyO, y+; *dNedd4*^T121FS^/TM3SerGFP |
| +; Sp/CyO, y+; *dNedd4*^T121FS^/TM3SerGFP (control) | | X | +; *Actin*-GAL4/CyO, y+; *dNedd4*^T121FS^/TM3SerGFP |

**Table S4**: Fly line crosses*

*All performed with replica lines to ensure the phenotypes are independent of integration sites.
