# Supplementary material for: A Splice Isoform of DNedd4, DNedd4-Long, Negatively Regulates Neuromuscular Synaptogenesis and Viability in Drosophila
Source: PLoS One. 2011 Nov 14;6(11):e27007. doi: 10.1371/journal.pone.0027007 (PMC3215714; doi:10.1371/journal.pone.0027007)
Supplement: Table S5 — Antibodies used for staining. (DOCX) [file pone.0027007.s008.docx]

**Table S5**: antibody staining

| Immunostaining | *Blocking** | 1^o^ antibody (dilution) | 2^o^ antibody (dilution) | Source of 1^o^ antibodies |
| --- | --- | --- | --- | --- |
| **3^rd^ instar Larval muscle and salivary gland** | | | | |
| Comm-ECD | 2%BSA+5%DS | Anti Comm-ECD (1:50) | Donkey anti rabbit-Cy3 (1:500) | G. Tear |
| DraQ5 |  | DraQ5 (1:500) |  | eBioscience |
| Flag | 2%BSA+2%NGS | Anti Flag (1:800) | Goat anti rabbit- Alexa Fluor^488^ (1:1000) | Cell Signaling |
| HRP | 2%BSA+2%NGS | Anti HRP-Cy3 (1:400) |  | ICN Pharmaceuticals, Inc. |
| Phalloidin | 2%BSA+2%NGS | Phalloidin (anti- F-actin) - Alexa Fluor^488^ (1:75) |  | Invitrogen |
| **S2 cells** | | | | |
| Flag | 2%NGS | Anti Flag(1:1000) | Goat anti mouse-Cy3 (1:1000) | Sigma |
| **Western blotting#** | | | | |
| actin | 5% dry milk | Anti actin, JLA20 (1:250) | Anti mouse HRP (1:10,000) | Iowa University |
| Comm-HA | 10% skim milk | Anti HA* | Anti mouse HRP | Covance |
|  |  | (1:10,000) | (1:10,000) |  |
| Flag | 5% dry milk | Anti Flag (1:10,000) | Anti mouse HRP (1:10,000) | Sigma |
| GST | 5% dry milk | Anti GST (1:5000) | Anti mouse HRP (1:10,000) | Covance |
| ubiquitin | 3% skim milk | Anti ubiquitin (1:1000) | Anti mouse HRP (1:10,000) | Babco |

*Abbreviations: DS=Donkey serum; NGS=Normal Goat Serum; BSA=Bovine Serum Albumin;

HA, hemagglutinin.

# Western blotting (immunoblotting) was performed on cells or tissues lysed in Lysis buffer plus protease inhibitors, as described in the Materials and Methods.
